# Influence of Warning Bleeding on Blood Loss in Low-Lying Placenta

**DOI:** 10.7759/cureus.74858

**Published:** 2024-11-30

**Authors:** Hiroto Yamamoto, Kaoru Yamawaki, Kazufumi Haino, Kosuke Yoshihara, Koji Nishijima

**Affiliations:** 1 Department of Obstetrics and Gynecology, Niigata University Medical and Dental Hospital, Niigata, JPN

**Keywords:** blood loss, cesarean section, childbirth, low-lying placenta, obstetric, obstetric labor complications, placenta, trial of labor, uterine hemorrhage, warning bleeding

## Abstract

Objective

This study aimed to investigate whether the amount of blood loss during delivery in patients with low-lying placenta is affected by the planned mode of delivery, internal os distance, and warning bleeding.

Materials and methods

We conducted a single-center retrospective study encompassing women with singleton pregnancies diagnosed with low-lying placenta between January 2012 and December 2021. Data for maternal demographic details and pregnancy outcomes were extracted from the institution's records. We analyzed blood loss during delivery according to the planned delivery mode, internal os distance (≥10 mm or within 10 mm), and the occurrence of warning bleeding. We also assessed the frequency of abnormal hemorrhage at delivery. Statistical analyses included the Mann-Whitney U test and Fisher's exact probability test, with significance set at p<0.05.

Results

This study included 27 pregnant women. The planned delivery mode and internal os distance showed no statistically significant impact on the amount of blood loss or frequency of abnormal hemorrhage at delivery. However, the occurrence of warning bleeding had a significant effect on both factors.

Conclusion

In patients diagnosed with a low-lying placenta with warning bleeding, cautious delivery management is recommended owing to the possible increased risk of abnormal hemorrhage at delivery.

## Introduction

Low-lying placenta (LLP) is diagnosed when the distance between the placental edge and the internal os of the cervix is 20 mm or less [1,2]. The optimal mode of delivery in cases of LLP remains a matter of debate [3,4]. In clinical practice, it is largely determined according to the individual facilities, either through a trial of labor (TOL) or a scheduled cesarean delivery (CD).

According to some studies, scheduled CD is recommended in cases of LLP owing to the substantial risk of intrapartum hemorrhage, especially when the internal os distance (IOD) is ≤10 mm [1,4,5]. Wortman et al. found that pregnancies with LLP were at risk for significant bleeding complications while they reported that the placenta-OS distance did not significantly affect bleeding outcomes (antepartum bleeding, cesarean due to bleeding, estimated blood loss at delivery, or postpartum hemorrhage) [6].

Moreover, a few studies have focused on the correlation between the amount of blood loss at delivery and antepartum bleeding in cases of LLP. Antepartum bleeding in placenta previa is sometimes referred to as "warning bleeding" [7,8]. According to the conventional definition, LLP is included in placenta previa [8-10]. Jean CA et al. defined antepartum bleeding of placenta previa (including LLP) as a form of warning bleeding, which they attributed to the difference in the growth rates of the lower uterine segment and placenta during the third trimester [8].

This study aimed to determine the effects of the planned mode of delivery, IOD, and warning bleeding on the amount of blood loss during delivery in cases of LLP.

## Materials and methods

We conducted a retrospective study of women with singleton pregnancies who delivered at our institution between January 2012 and December 2021.

LLP was diagnosed if the IOD, which is the distance between the placental edge and the internal os of the cervix, was ≤20 mm [1,2]. Women with singleton pregnancies diagnosed as having LLP on transvaginal sonography at 35-36 weeks of gestation were included. The exclusion criteria were the presence of placenta previa, antenatally suspected placenta accreta spectrum, and multiple pregnancies. Additionally, we excluded pregnant women receiving anticoagulation or antiplatelet therapy, and those with abnormal platelet counts or coagulation systems.

Maternal demographic characteristics and pregnancy outcome data were extracted from the medical records at our institution. The collected data included information regarding maternal age, past pregnancy, body mass index, planned mode of delivery, labor progress, actual delivery mode, indications for intrapartum interventions, period of hospitalization, blood transfusion, hemostatic surgery, maternal mortality, the occurrence of antepartum bleeding (warning bleeding), and amount of blood loss at delivery. For vaginal deliveries, we measured the blood loss from the onset of labor to 2 h after delivery. For the CDs, we measured the total intraoperative blood loss. For hemostasis, various procedures, such as compression hemostasis, suturing, administration of uterine contractile agents, and placement of uterine hemostatic balloons, were occasionally performed. We also collected data on neonatal outcomes (birth weight, 1-min Apgar score, 5-min Apgar score, umbilical cord arterial pH, and neonatal mortality). Warning bleeding was defined as any bleeding from within the uterus during pregnancy that required hospitalization and occurred without any associated uterine contractions or abdominal pain. Our institution has no clear policy regarding the mode of delivery in cases of LLP. Thus, the decision was made at the discretion of the attending physician and the patient's request, based on transvaginal ultrasound findings and other perinatal information.

We analyzed the blood loss at delivery in relation to the planned mode of delivery, IOD (≥10 mm or within 10 mm), and the occurrence of warning bleeding. We also assessed the frequency of abnormal hemorrhage at delivery to compensate for the changes in the amount of blood loss due to differences in the mode of delivery. Abnormal hemorrhage at delivery was defined as bleeding of 800 mL or more in vaginal delivery and 1500 mL or more in CDs, following the 90th percentile of perinatal hemorrhage, according to the Perinatal Committee of the Japanese Society of Obstetrics and Gynecology.

Statistical analyses were performed using the Mann-Whitney U test and Fisher's direct probability test. Statistical significance was set at p < 0.05. All statistical analyses were performed using the GraphPad Prism 8 software (GraphPad Software Inc., San Diego, CA, USA). All procedures were performed in accordance with the provisions of the Declaration of Helsinki in 1964 and later versions. Ethical approval was obtained from the Research Ethics Board of Niigata University (approval number 2022-0278). Due to the retrospective nature of the study, an opt-out approach was followed instead of obtaining patient consent.

## Results

During the study period, our institution handled 4465 deliveries, which included 31 women with confirmed LLP on transvaginal sonography at 35-36 weeks of gestation. Four women did not meet the eligibility criteria and were excluded (two had a confirmed diagnosis of marginal placenta previa, one had suspected placenta accreta spectrum, and one had multiple pregnancies). Thus, 27 pregnancies were included in the analysis.

**Figure 1:**
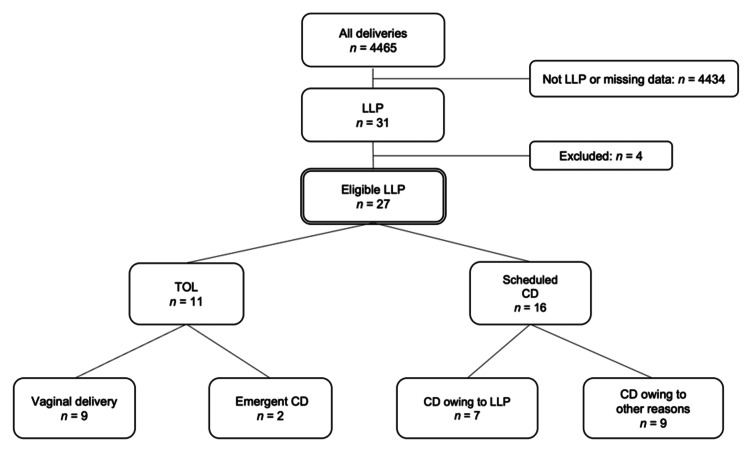
Mode of delivery in 27 patients with LLP LLP, low-lying placenta; CD, cesarean delivery; TOL, trial of labor

Maternal demographic characteristics and pregnancy outcomes are detailed in Table 1, and the pregnancy course and delivery outcomes are illustrated in Figure 1. Eleven women (41%) underwent a TOL, of which 9 (82%) achieved vaginal delivery. In the two remaining patients who underwent a TOL, emergent CD was performed because of labor arrest. None of the cases required emergent CD due to antepartum or intrapartum hemorrhage. Sixteen women (59%) with LLP underwent scheduled CD. Among them, seven (44%) underwent CD solely for LLP; the other indications included previous CD (n = 5), severe gestational hypertension (n = 1), fetal disease (n = 1), marginal sinus (n = 1), and vasa previa (n = 1). Subsequently, we analyzed the amount of blood loss during delivery.

**Table 1:** Maternal background and infant outcomes Data are presented as median (range) or n (%).

Characteristics		n = 27
Maternal age (y)		34 (24–43)
Nulliparous		16 (59)
Multiparous		11 (41)
Gestational age at delivery (weeks)		38 (37–41)
Neonatal birth weight (g)		2994 (2088–3826)
1-min Apgar score		8 (7–9)
5-min Apgar score		9 (8–10)
Umbilical cord arterial pH		7.32 (7.12–7.44)
Placental location: Anterior		2 (7)
Placental location: Posterior		25 (93)

Comparison based on the planned mode of delivery

**Figure 2:**
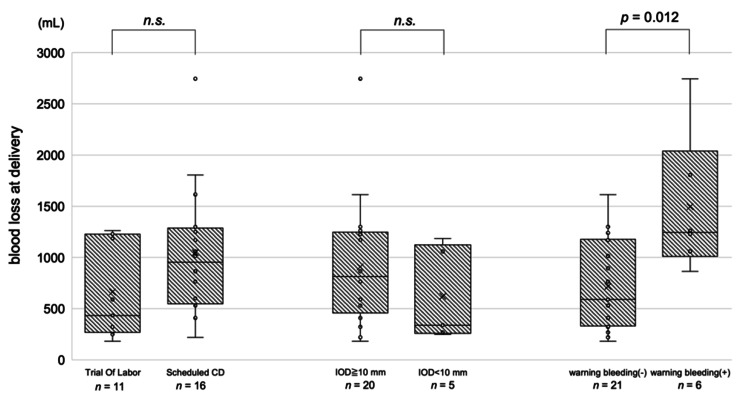
Amount of blood loss at delivery in each group. Note: Mann–Whitney U test. n.s., not significant; IOD, internal os distance; CD, cesarean delivery.

Maternal demographic characteristics and pregnancy outcomes for the TOL and scheduled CD groups are presented in Table 2. A scheduled CD was performed at less than 39 weeks of gestation, and the two groups showed significant differences in the number of weeks of gestation and birth weight of the infants. No significant intergroup differences were observed in maternal age, delivery history, or infant outcomes except birth weight. The amount of blood loss at delivery in both groups is shown in Figure 2. The median (range) blood loss at delivery was 433 mL (182-1263 mL) in the TOL group and 955 mL (220-2745 mL) in the scheduled CD group (p = 0.33).

**Table 2:** Comparison of maternal background, infant outcomes, and results Note: Data are presented as median (range) or n (%). *Statistically significant difference (p<0.05) CD, cesarean delivery; IOD, internal os distance; BMI, body mass index; NA, not applicable

	Trial of labor			Scheduled CD			U-value	p-value		IOD ≧10 mm			IOD <10 mm			U-value	p-value		Warning bleeding (-)			Warning bleeding (+)			U-value	p-value
	(n = 11)			(n = 16)						(n = 20)			(n = 5)						(n = 21)			(n = 6)				
Characteristics																										
Maternal age (y)	35	(28–41)		33	(24–43)		85.5	0.90		32.5	(24–43)		35	(29–41)		48.5	0.92		35	(28–43)		29	(24–41)		41	0.20
Parity	0	(0–1)		0	(0–3)		79.5	0.63		0	(0–3)		0	(0–1)		49	0.94		0	(0–3)		0	(0–1)		43	0.18
BMI (kg/m^2^)	23.4	(21.1–30.1)		24.3	(19.8–35.4)		84	0.87		24.7	(19.8–35.4)		22.8	(21.1–26.5)		35	0.34		23.4	(19.8–30.1)		25.8	(20.1–35.4)		46	0.35
Gestational age at delivery (weeks)	39	(38–41)		37	(37–38)		4	<0.01*		38	(37–41)		39	(37–41)		25.5	0.08		38	(37–41)		37	(37–39)		41.5	0.18
Neonatal birth weight (g)	3220	(2826–3826)		2838	(2088–3448)		23	<0.01*		2967	(2088–3826)		3056	(2866–3774)		30	0.17		2974	(2088–3826)		3186	(2660–3564)		44	0.27
1–min Apgar score	8	(7–8)		8	(7–9)		70.5	0.20		8	(7–9)		8	(8–8)		50	1.00		8	(7–9)		8	(8–9)		53.5	0.41
5–min Apgar score	9	(8–10)		9	(8–9)		73	0.27		9	(8–10)		9	(8–9)		45.5	0.66		9	(8–9)		9	(8–10)		55.5	0.52
Umbilical cord arterial pH	7.32	(7.20–7.44)		7.32	(7.12–7.38)		81.5	0.75		7.32	(7.23–7.44)		7.32	(7.20–7.42)		45	0.73		7.32	(7.20–7.44)		7.32	(7.12–7.35)		57.5	0.75
Outcome																										
Frequency of abnormal hemorrhage at delivery	4	(36.4)		3	(18.8)		NA	0.391		5	(25)		1	(20)		NA	1.000		3	(14.3)		4	(66.7)		NA	0.024*

Comparison based on IOD

Maternal demographic characteristics and pregnancy outcomes in the IOD ≥ 10 mm and IOD < 10 mm groups are presented in Table 2. Of the 27 patients analyzed, 2 with IOD < 2 cm at 35-36 weeks of gestation (where the distance was not clearly stated) were excluded from the analyses. The amount of blood loss at delivery in both groups is shown in Figure 2. The median (range) blood loss at delivery was 815 mL (182-2745 mL) in the IOD ≥ 10 mm group and 340 mL (250-1185 mL) in the IOD < 10 mm group (p = 0.22), with no significant difference between the two groups.

Comparison based on the occurrence of warning bleeding

We compared the group showing no significant genital bleeding during pregnancy with the group showing genital bleeding that required hospitalization and management during pregnancy. Maternal background and infant outcomes of both groups are shown in Table 2 and Table 3. The median (range) amount of blood loss at delivery was 590 mL (182-1300 mL) in the group with no warning bleeding and 1246 mL (865-2745) in the group with warning bleeding (Figure 2) and was significantly higher in the group with warning bleeding (p = 0.012).

**Table 3:** Summary of six patients with warning bleeding BMI, body mass index; G, gravity; P, parity; IOD, internal os distance; CD, cesarean delivery; VD, vaginal delivery; N.D., not detectable; LLP, low-lying placenta

No	Age (y)	BMI (kg/m^2^)	G	P	Planned mode of delivery	Actual delivery mode	IOD (mm)	Warning bleeding	Blood loss (ml)	Abnormal hemorrhage at delivery	Placental location	Gestational age at delivery (week)	Fetal presentation	Neonatal body weight (g)	1-min Apgar score	5-min Apgar score	Umbilical cord arterial pH	Remarks
1	40	24.5	2	0	CD	CD	N.D.	＋	1805	＋	posterior	37	cephalic	3152	9	9	7.12	marginal sinus+
2	24	20.7	1	0	CD	CD	11.5	＋	865	-	posterior	37	cephalic	2660	8	9	7.31	CD due to LLP
3	29	25.6	1	0	CD	CD	6.5	＋	1060	-	anterior	37	breech	2866	8	8	7.32	CD due to LLP
4	28	26	1	0	TOL	VD	10.1	＋	1229	＋	posterior	38	cephalic	3564	8	9	7.35	
5	29	27.9	3	0	TOL	VD	19.4	＋	1263	＋	anterior	39	cephalic	3220	8	10	7.31	
6	41	35.4	2	1	CD	CD	17.6	＋	2745	＋	posterior	37	cephalic	3448	8	9	7.32	CD due to previous CD

Table 2 also presents the data for the frequency of abnormal hemorrhage at delivery. This variable showed no significant difference between the TOL group and scheduled CD group nor between the IOD ≥ 10 mm group and the IOD < 10 mm group (p = 0.391, p = 1.000, respectively). However, the frequency of abnormal hemorrhage at delivery was significantly greater in the warning bleeding group (4/6 (66.7%)) than in the group with no warning bleeding (3/21 (14.2%)) (p = 0.024).

None of the 27 women analyzed in this study underwent obstetric hysterectomy, maternal death, or neonatal death.

## Discussion

In this study, we conclusively demonstrated that the presence of warning bleeding was associated with a notable increase in the amount of blood loss at delivery. Although previous studies have explored the impact of warning bleeding in LLP on the rates of emergent CD necessitated by hemorrhage [6,11], scant attention has been directed toward assessing its influence on the actual quantity of blood loss during delivery.

Warning bleeding is sometimes defined as painless genital bleeding from the placenta [8]. However, a precise definition of this phenomenon is difficult. We attempted to characterize it as a type of bleeding distinct from placental abruption and signs of labor, which often include uterine contractions, by defining it as described earlier. Several reports underline the importance of warning bleeding in assessing the risks of adverse pregnancy outcomes [12,13]. However, to date, the extent to which warning bleeding contributes to overall blood loss during delivery has been unclear. Our study provides evidence that warning bleeding before delivery significantly augments both total blood loss at delivery and the incidence of abnormal hemorrhage during delivery.

The small number of cases in this study made it difficult to differentiate between the actual modes of delivery when comparing the amount of blood loss in each group. Instead, we compared the frequency of abnormal bleeding during delivery in each mode and considered the impact of each factor on the amount of blood loss. Numerous studies have investigated the factors that predict blood loss in LLP deliveries. Although the rates of CD increase with a shorter IOD, sporadic reports suggest that vaginal delivery may be safely attempted when the distance exceeds 10 mm [3,14,15]. Conversely, some Japanese studies have advocated the safety of vaginal delivery regardless of the IOD [16-18]. Notably, in our study, neither the quantity of blood loss at delivery nor the frequency of abnormal bleeding during delivery significantly increased in the group with IOD < 10 mm. Consequently, if LLP is diagnosed via transvaginal sonography at 35-36 weeks, vaginal delivery may be considered a viable option, even with an IOD of <10 mm.

As an additional strength of our investigation, women diagnosed with LLP who presented with warning bleeding were also considered eligible for TOL. Although instances of such cases were limited, successful vaginal delivery was achieved in these cases. Furthermore, the eligibility criteria were refined to encompass only women diagnosed with LLP between weeks 35 and 36 of gestation. In prior studies addressing LLP and placenta previa, the impact of placental migration has been described [19,20]. Our deliberate selection criteria substantially mitigated the potential influence of placental migration.

This study had some limitations. The primary limitation of our cohort study was its retrospective design, which potentially led to missing data and data entry errors. The variations in transvaginal ultrasound images used to diagnose LLP represented another limitation. The absence of a fixed policy for perinatal management of LLP at our institution, coupled with the discretionary decision-making process regarding vaginal delivery, were also sources of potential biases. The choices of the attending physicians and patients are influenced by the perinatal information available to them. This was reflected in the relatively high percentage (25.9%) of CDs solely for LLP, where factors such as IOD and the occurrence of warning bleeding may have influenced the decision-making process. Furthermore, the cause of the bleeding was unclear. While we defined it as bleeding caused by the LLP itself, some of the bleeding could have involved signs of labor. Lastly, the single-center nature of this study may limit the generalizability of the findings owing to the infrequency of cases defined as LLP.

Watanabe et al. reported that blood loss during CD was higher in cases of placenta previa without warning bleeding, where tight attachment was considered relevant [7]. We speculate that while blood loss in cases of placenta previa covering the internal os may be strongly related to placental changes toward the placenta accreta spectrum, this association may be less significant in cases of LLP. The mechanism underlying warning bleeding in LLP remains unclear. We consider the mechanism to be similar to that of placenta previa covering the internal os and recognize the origin of the bleeding to be between the placenta and myometrium. Patients with LLP that causes warning bleeding are prone to small detachments between the placenta and myometrium, which may impede the hemostatic mechanism after placental detachment.

## Conclusions

Our study demonstrated that in pregnant women diagnosed with LLP at 35-36 weeks of gestation, neither the planned mode of delivery nor the IOD significantly influenced the amount of blood loss at delivery or the frequency of abnormal hemorrhage. However, the presence of warning bleeding was associated with increased blood loss at delivery and a higher frequency of abnormal hemorrhage. These findings underscore the importance of rigorous prepartum preparation for patients with LLP and warning bleeding due to their heightened risk of excessive blood loss.
